# Carnot and Joule Cycles Implied in Generalized Entropy and Exergy Properties as Fundamentals of Extrema Principles

**DOI:** 10.3390/e27121219

**Published:** 2025-11-29

**Authors:** Pierfrancesco Palazzo

**Affiliations:** Technip Energies, 00148 Rome, Italy; pierfrancesco.palazzo@ten.com or pierfrancesco.palazzo@gmail.com

**Keywords:** Carnot cycle, Joule cycle, generalized entropy, generalized exergy, non-equilibrium, entropy production, entropy generation, complexity, self-organization, constructal theory, autopoiesis

## Abstract

Thermodynamic laws and principles overarch all domains of physics, chemistry and biology. In this broad perspective, further generalizations with respect to the “state-of-the-art” of current theories are still viable considering all aspects of systems’ states and phenomena. This research aims to discuss the physical and informational implications of Carnot and Joule cycles and the properties inferred from their definitions, as well as the extrema principles governing non-equilibrium phenomena in complex systems. The approach adopted can be viewed as an analytical variational method focusing cycles’ performances and maxima of properties and parameters along cyclic processes. The dualisms and symmetries characterizing Carnot and Joule cycles imply the inference of the necessity and sufficiency of the stable equilibrium for equality of thermodynamic potentials of any system in any state. The conclusions provide a perspective on complex systems and non-equilibrium processes governed by the extrema principles and the physical and informational properties relating to complexity and self-organization. This treatise also represents a basis and a proposal for further developments looking forward to unifying thermodynamic and informational aspects of extrema principles in the direction of complexity, self-organization, constructal emergence and autopoiesis of non-living and living systems in the frame of a general unifying paradigm. Implications and applications are envisaged in an extended perspective accounting for sustainability, circularity and biotechnologies representing future scenarios of industry and environmental protection.

## 1. Introduction

During recent decades, in the framework of Thermodynamics and fundamental principles, First and Second Law statements have been re-established by Hatsopoulos and Gyftopoulos [1,2,3,4] with an axiomatic paradigm in a different perspective positing the following postulates: (i) energy is a state property of any system in any state, this statement being assumed as an enunciate of the First Law; (ii) stable equilibrium exists and is unique, that constituting an essential aspect of the Second Law. In this perspective, the concept of entropy results as an inherent property of matter–energy entity and has been defined as a corollary inferred from the difference in energy and available energy, and the non-existence of a Perpetual Motion Machine of the Second Kind (PMM2) [5]. Nevertheless, the first historical definitions of entropy, conceived by Carnot and Clausius, remain valid expressions implied with theoretical developments and applications. The thermodynamic potentials, representing “driving forces” of phenomena and processes occurring in any system, deserve a generalization of current definitions and their relationship with the stable equilibrium, or non-equilibrium, and with the correlated definition of the entropy property as a corollary of the Second Law [6,7]. Indeed, the equality of potentials is assumed as a solely necessary condition for mutual stable equilibrium between two interacting systems [5]. Instead, this relationship needs to become bi-univocal by means of both necessity and sufficiency as logical co-existing implications between the two thermodynamic conditions, that is, stable equilibrium and potentials equality, characterizing thermodynamic states.

Arguing on the concepts leading “from applications to fundamentals”, it could be regarded as the logical syllogism of inductive inference, that steps up from lower to higher hierarchical levels, from particular cases to general abstraction. In effect, this line of reasoning often occurs along the repetitive iterations based on inductive and/or deductive inference. Scientific methods progress through construction processes of ever more generalized paradigms. Thermodynamics, in its widest perspective, is a domain where it is still allowed a generalization of concepts pertaining to foundations moved by the need of a rigorous and rational methodology to evaluate systems, phenomena and applications. The outset of the present discussion is firstly represented by a reconsideration of the internal energy schema and the Gibbs–Duhem Relation. Moreover, a theoretical variational analysis of Carnot and Joule cycles is proposed, in line with recent developments by Feidt with a theoretical finite-time approach to non-equilibrium processes finalized to applications [8,9,10,11]. Subsequently, fundamentals of the MIT thought are discussed and the generalized bi-univocal relationship above mentioned is explained. Finally, an overarching standpoint on complexity, self-organization, constructal theory of non-living systems and autopoiesis of living organisms is proposed.

## 2. Axioms and Methods

The rationale underpinning the paradigm and subsequent conclusions of this research is based on two axioms: (i) generalizing the physical meaning and logical sense of reasoning needed to account for all contributions to phenomena, and: (ii) the degree of generality and robustness of a demonstration imply the proof of both necessity and sufficiency of conditions for a complete and consistent derivation of the thesis.

The adoption of an ideal gas as a model to represent a multi-particle system, and the framework of the Kinetic Theory of Gases, as a set of phenomena fundamental to Statistical Mechanics and Statistical Thermodynamics, does not represent a mere simplification of a real system to posit assumptions and derive conclusions. Instead, the model of ideal systems is adopted to demonstrate that, even in the particular case of ideal conditions, phenomena occur, and properties behave, with similar or even the same behaviors as those of real systems and are corroborated by real phenomena and processes.

As far as the frame of fundamental laws of thermodynamics are concerned, the First Law is enunciated in terms of the existence of energy as a state property [5]. The Second Law, according to the same line of thought, is enunciated as a statement of existence and uniqueness of stable equilibrium from which the non-existence of a Perpetual Motion Machine of the Second Kind (PMM2) is used to prove the formulation of the entropy property according to Gyftopoulos and Beretta [5] and the school of thought of MIT.

A caveat is here clarified pertaining to the physical entities mass, heat and work interaction that represent concepts introducing a logical loop in the definition of thermodynamic properties. Although mass, heat and work fluxes are not exact differentials, and derive as mass and energy transfer, those interactions are mentioned in the present treatise to identify transmissions through systems boundaries [12,13,14,15].

The analysis of the conceptual schema of energy property and related contributions, characterizing any system in any state, is here firstly proposed to attempt a further generalization of fundamental relations to overarch all energies and related forms concurring to the internal energy of a portion of matter interacting with the external environment.

## 3. Internal Energy Schema and Related Components

Any physical system is inherently characterized by a content of mass and related internal energy defined by intensive properties, or thermodynamic potentials, and extensive properties. The existence of the energy property is considered an axiom and is expressed in the statement of the First Law [5]. The Gibbs–Duhem Relation establishes a constraint among all intensive properties constituting thermodynamic potentials of heterogeneous systems composed by a given number of chemical constituents:

(1) $$SdT+\sum_{i=1}^{r}n_{i}d\mu_{i}-VdP=0$$

in which r indicates the number of chemical constituents of the system, $n_{i}$ (number of moles) and $\mu_{i}$ (chemical potentials) refer to each and every chemical substance composing the system.

As far as thermodynamic potentials are concerned, chemical potentials $\mu_{i}$ are defined as the components of internal energy generated by the interactions determined by inter-particle positions and relative distances and “entanglements” among particles and/or interactions depending on relative particle configurations such as, for instance, chemical bonds among atoms and molecules. Instead, kinetic potentials are due to inter-particle relative velocities and relative vectorial components.

In the frame of the Kinetic Theory of Gases, as a fundamental basis of Classical Statistical Mechanics and Classical Statistical Thermodynamics, the whole content of internal energy, characterizing any real multi-particle system, derives from the contribution of energy associated with each and every constituting particle. Hence, the internal energy is directly correlated to the kinetic energy, depending on the relative velocity of particles, and to the potential energy depending on the relative position of particles and the configuration of atoms and/or molecules aggregates determined by chemical bonds. Instead, pressure is the result of the frequency of collisions due to the particles’ velocity and kinetic energy, and of the intensity of interactions due to the particles’ position and potential energy, among particles and the wall of the external system, such as a cylinder-piston device or a closed vessel. The consequence is that pressure is always associated with temperature and/or to chemical potentials and is dependent on those thermodynamic potentials through the thermal State Equation $PV=n\bar{R}T$ or the chemical State Equation $PV=n\bar{R}\mu$ [16].

The Gibbs–Duhem Relation is affected by an inconsistency appearing in the special case of an ideal gas with a fixed chemical composition for which temperature and pressure are the thermodynamic potentials governing the process and determining the internal energy U. In particular, temperature is the inter-particle kinetic energy, transformed into inter-particle potential energy (Lennard-Jones potential) due to repulsive interactions at any collisions (attractive interactions are negligible in the ideal gas model), with no physical interactions or chemical reactions occurring inside the system as assumed.

In case of an isothermal process of an ideal gas, the chemical potential remains constant; indeed, by definition of chemical potential $\mu_{i}={\left(\frac{\partial U}{\partial n_{i}}\right)}_{S,n_{j\neq i},V}$ hence $d\mu_{i}=0$; in fact, considering that the internal energy U of the assumed ideal gas depends on temperature only that, in turn, is constant along the isothermal process, hence U is constant, ∂U is null (regardless the variation of $S,n_{j\neq 1},V$), and the chemical potentials $\mu_{i}$ are constant, thus $d\mu_{i}=0$; as above pointed out, from a physical standpoint, for the assumed ideal system, the only contribution to the chemical potentials $\mu_{i}$ derives from the transformation, at any collision, of the particles’ kinetic energy, associated with the temperature, into the particles’ potential energy (Lennard-Jones), associated with the chemical potentials; being the temperature constant, the chemical potentials are constant as well; therefore, the Gibbs–Duhem Relation reduces to: SdT=VdP, where S and V are not null, both being inherent properties of any system in any state.

In case of an isopotential process, with no thermal variations occurring, the temperature remains constant, hence dT=0 and the Gibbs–Duhem Relation reduces to $\sum_{i=1}^{r}S_{i}^{C}{d\mu }_{i}=VdP$. These two simplified Gibbs–Duhem Relations should remain valid along an isothermal or isopotential reversible process, with the temperature, or chemical potentials remaining constant by definition, so that dT=0 or ${d\mu }_{i}=0$, respectively; nevertheless, dP undergoes variations along the same isothermal or isopotential processes while the volume is inherently not null; therefore, an inconsistency appears in both previous simplified Gibbs–Duhem Relations where the left side member is null, and the right side is not null. Moreover, if an isobaric process is considered, the pressure remains constant and dP=0 while temperature, or chemical potentials, changes; this implies that for isobaric processes, a similar inconsistency is demonstrated with respect to the previous cases of isothermal or isopotential processes. The variation in temperature dT and/or the variation in chemical potential $d\mu_{i}$, needed to ensure the validity of the (thermal or chemical) Gibbs–Duhem Relation against the variation in pressure dP, implies that (thermal or chemical) entropy property must change along isothermal and/or isopotential processes, respectively, and, as a consequence, temperature and/or the chemical potential are not constant, which is in contradiction with the assumption set forth by the definition of isothermal and isopotential processes. The temperature and/or chemical potentials appearing in any system are caused by attractive and repulsive interactions, occurring at collisions, and depends on molecules’ kinetic energy associated with their velocity, and potential energy associated with their positions, so that temperature and/or chemical potential constitute the first contribution to the pressure due to the frequency or density of collisions. The second contribution is ascribed to the (specific) volume determining the density of the internal system interactions and again the frequency and intensity of collisions between molecules and the boundary wall of the external system.

The Gibbs–Duhem inconsistency pointed out above can be resolved through a generalized definition of properties and equations determined by physical systems phenomena and processes. Gibbs Relation and Euler Relation are expressed by means of generalized properties as here after described.

The (thermal) Gibbs Relation expressing the First Law and valid for the particular case of ideal gases dU=TdS−PdV=δQ+δW can be reformulated in different terms introducing the definitions of thermal entropy $S^{T}$ and mechanical entropy $S^{M}$ [17,18]:

(2) $$\mathrm{dU}\;=\;T\left({dS}^{T}\right)-\frac{PV}{\bar{R}}\left(dS^{M}\right)=\delta Q+\delta W$$

that, by using the state equation $PV=\bar{R}T$, valid in this special case of ideal systems, becomes in the following form:

(3) $$\mathrm{dU}\;=\;T\left({dS}^{T}\right)-T\left(dS^{M}\right)=T\left({dS}^{T}-dS^{M}\right)=\delta Q+\delta W$$

This expression of (thermal) Gibbs Relation, associated with the temperature, takes into account either elementary heat interaction δQ or elementary work interaction δW contributing to infinitesimal variations in internal energy dU that remains constant along an isothermal process considered so far.

As an alternative, the Gibbs Relation used in the above reasoning can be expressed on the basis of the chemical potentials $\mu_{i}$, instead of temperature, and the elementary mass interaction δM, hence:

(4) $$dU=\sum_{i=1}^{r}\mu_{i}\left(dS_{i}^{C}\right)-\frac{PV}{\bar{R}}\left(dS^{M}\right)=\delta M+\delta W$$

and transformed by using the state equation $PV=\sum_{i=1}^{r}\bar{R}\mu_{i}$ [16], valid in this special case of ideal systems, in the following form of the (chemical) Gibbs Relation:

(5) $$\mathrm{dU}\;=\;\sum_{i=1}^{r}\mu_{i}\left(dS_{i}^{C}\right)-\sum_{i=1}^{r}\mu_{i}\left(dS^{M}\right)=\sum_{i=1}^{r}\mu_{i}\left(dS_{i}^{C}-dS_{i}^{M}\right)=\delta M+\delta W$$

If conversion cycles are considered, and assuming that the operating fluid is a real gas where inter-particle kinetic and potential interactions are not null, isothermal or isopotential processes, along thermal or chemical Carnot cycle, imply energy transformation and a corresponding entropy transformation. Instead, non-heat-interaction (adiabatic) and non-mass-interaction (non-permeable) processes, or isovolumic processes (non-work), imply energy conversion and a corresponding entropy conversion.

In case the system is assumed as ideal and characterized by the kinetic energy of particles and no potential energy thereof, the adiabatic reversible (isoentropic) compression or expansion process consists of a work interaction determining a transfer of macroscopic work PdV into microscopic inter-particle kinetic energy and a subsequent increase or decrease in temperature; hence a non-cyclic conversion (low-pressure to high-pressure and low-temperature to high-temperature, or opposite) occurs. The isovolumic heating process consists of a heat interaction implying a transfer of macroscopic heat ${TdS}^{T}$ into microscopic inter-particle kinetic energy determining the increase in pressure; also in this case, a non-cyclic conversion (low-temperature to high-temperature and low-pressure to high-pressure, or opposite) occurs. A similar reasoning is applicable to ideal systems with a prevailing contribution of potential energy due to inter-particle interactions and related chemical internal energy, with respect to kinetic energy of particles and related thermal internal energy assumed as negligible.

For an ideal system, for which internal energy is constituted by thermal energy, the chemical potentials appear solely as a consequence of particles’ kinetic energy associated with velocity, so that constant temperature along an isothermal process above considered constitutes the main contribution to the pressure. The additional contribution is due to the (specific) volume determining the density, and consequently the frequency, of collisions among particles and the containing wall of the external system that can be open or closed, as in the special case of a cylinder-piston device. The density, instead, could not determine the chemical potentials by means of interactions depending on relative positions simply because inter-particle interactions are assumed as negligible and repulsive interactions only appear at inter-particle collisions.

In case the component of internal energy is the chemical energy, the thermal potential derives from transformation of molecules’ potential energy associated with positions. Consequently, chemical potentials constitute the sole contribution to pressure by means of Van der Waals attractive or repulsive interactions, or by chemical bonds of atomic–molecular aggregates. The additional contribution to the pressure is due to the (specific) volume or density determining the intensity of interactions among particles and the containing device. The density, in this case, could not determine the thermal potential being inter-particle kinetic interactions negligible.

The Euler Relation expresses in finite terms the amount of total, or generalized, internal energy (of the “permanent” system) and the total, or generalized, external energy (of the “transiting” system) with thermal, chemical and mechanical contributions:

(6) $$U^{G}=U^{G}\left(T,\mu_{i},P\right)=U^{T}\left(T\right)+U^{C}\left(\mu_{i}\right)+U^{M}\left(P\right)=TS^{T}+\sum_{i=1}^{r}\mu_{i}S_{i}^{C}-PV$$

The physical meaning of the entropy property addresses to the degree of subdivision of any phenomena among the constituting elements of a system. Indeed, entropy depends on the thermodynamic potential and, along with an isothermal (or isopotential) process, the temperature (or the chemical potential) remains constant while a heat interaction (or mass interaction) occurs; the variation in the entropy property characterizes the thermal energy transfer from the external to the internal system by means of heat interaction and volume variation. Thus, the degree of distribution of thermal energy inside the “permanent” system, delimited by the control surface, does not remain constant since the kinetic energy associated with each and every particle remains constant as the temperature is constant, while the thermal entropy changes with the volume since the thermal entropy undergoes variation due to volume variations. For this very reason, the “transiting” thermal energy at constant temperature is associated with a thermal entropy input, or output, through the control surface of the system while the entropy property increases or decreases depending on volume variation in the transiting mass. This fact is due to the dependence of the thermal internal energy on temperature and the thermal entropy associated with both temperature and volume. Hence, the transiting thermal entropy flow would change the content of the permanent system internal energy that instead should remain constant while undergoing an isothermal elaboration equivalent to a cyclic process for which Q=W. Therefore, heat interaction input must be balanced by the work interaction output (or vice versa) and, consequently, thermal entropy associated with heat interaction must be compensated by an equal amount of mechanical entropy that, instead, has to be associated with the work interaction to achieve the overall entropy property balance: $S^{T}=S^{M}$. A similar argument can be applied to isopotential processes for which M=W and consequently $S^{C}=S^{M}$. This is the proof that entropy is an inherent property of all systems in all states, and it is associated with all types of interactions (thermal, chemical and mechanical), between two systems.

The balance of entropy, along the isothermal reversible process with dU=0, is deduced from Equation (3), that is, ${dS}^{GENERALIZED}={dS}^{G}={dS}^{T}-dS^{M}=0$ where $dS^{T}$ is the thermal entropy input or output, due to heat interaction, and $-dS^{M}$ is the mechanical entropy output or input, respectively, due to work interaction. The physical meaning is that the transformation from the kinetic form of thermal entropy into the geometric form of mechanical entropy, required to transform (not to convert) heat into work (or vice versa), needs a variation in the mechanical entropy content characterizing the mechanical internal energy $U^{M}=-PV$ remaining within the system since $dU=dU^{T}\left(T\right)+dU^{C}\left(\mu_{i}\right)+dU^{M}\left(P\right)=0$.

As the mechanical entropy must enter the system because of work output, then the mechanical entropy flow direction is inverted compared to the thermal entropy flow direction in the system’s entropy balance. The generalized entropy is consequently constant if both thermal entropy and mechanical entropy enter, or exit, the system since both entropy components have an opposite origin and elide with each other. Hence, the generalized entropy, resulting from the additive contributions of thermal and mechanical components of entropy, implies that thermal entropy is not constant in an isothermal reversible process that requires heat interaction by means of thermal entropy flow. Instead, the pressure does not represent an independent thermodynamic potential, and it is just the mechanical effect produced by the temperature itself; pressure is therefore an apparent thermodynamic potential that, by virtue of its dependence on temperature, is constrained by the (thermal) State Equation. A similar rationale is applicable to isopotential processes starting from Equation (5) in which the chemical potential replaces the temperature.

Though, being directly correlated to the temperature characterized by thermal entropy, or to the chemical potentials characterized by chemical entropy, the pressure has somehow a similar behavior and is in turn characterized by its own entropy assuming a mechanical definition depending on the geometry of the system. In this regard, the Thermal State Equation, or the Chemical State Equation, establish a relationship between the temperature, or the chemical potentials, and the mechanical internal energy; nevertheless, pressure and specific volume (or density) can vary independently; this means that, notwithstanding the mechanical internal energy depends on the thermal internal energy, the pressure and the mechanical entropy are independent because of the system density, while the mechanical internal energy remains constant. Hence, the mechanical entropy ends up being similar to thermal entropy and chemical entropy and should be accounted for in the generalized entropy associated with the generalized internal energy of the “permanent” system and to the flow contribution of the “transiting” system through the control surface. Finally, since the mechanical entropy is directly correlated to the pressure, it must be considered as a component of the generalized entropy, in addition to thermal entropy and chemical entropy inherently characterizing any system in any state.

The Gibbs Relation, using the generalized form of the Euler Relation reported above, resolves the apparent inconsistency highlighted in the canonical form of the Gibbs–Duhem Relation expressed in Equation (1). Indeed, for a real system, the Gibbs Relation undergoes the following change in formal expression introducing the mechanical entropy $S^{M}$ according to Equations (3) and (5):

(7) $$dU=T\left(dS^{T}-dS^{M}\right)+\sum_{i=1}^{r}\mu_{i}\left(dS_{i}^{C}-dS_{i}^{M}\right)=\delta Q+\delta M+\delta W\;dU=TdS^{T}-TdS^{M}+\sum_{i=1}^{r}\mu_{i}dS_{i}^{C}-\sum_{i=1}^{r}\mu_{i}dS_{i}^{M}=\delta Q+\delta M+\delta W$$

The Euler Relation is obtained from the Gibbs Relation by integration at constant temperature and constant chemical potential [1], so that:

(8) $$U=T\left(S^{T}-S^{M}\right)+\sum_{i=1}^{r}\mu_{i}\left(S_{i}^{C}-S_{i}^{M}\right)=Q+M+W\;U=TS^{G,T}+\sum_{i=1}^{r}\mu_{i}S_{i}^{G,C}=Q+M+W$$

The canonical Euler Relation, compared with the above generalized version expressed by means of the components of entropy, implies that the term −PV corresponds and relates to the terms $-TS^{M}$ and $\mu_{i}S_{j}^{M}$. Assuming, for example, an isothermal process with no chemical reactions (so that chemical potential remains constant), the thermal entropy variation is equal to the mechanical entropy variation (as both depend on volume variation). In the case of the ideal system as assumed, the internal energy U is associated solely to the particles’ kinetic energy and thus to the temperature only; however, internal energy components also depend on the terms $\sum_{i=1}^{r}\mu_{i}n_{i}$ and −PV which both depend on the volume as well. This dependency ensures that real systems, in which thermodynamic conditions are affected by interactions among molecules that determine the potential energy, are characterized by the volume which affects the mean distance among particles and therefore the potential generated by the inter-particle actions.

The Euler Relation expressed in Equation (8) by specific and generalized thermodynamic potentials becomes, in differential terms:

(9) $$dU=TdS^{G}+S^{G}dT+\sum_{i=1}^{r}\mu_{i}dS_{i}^{G}+\sum_{i=1}^{r}S_{i}^{G}d\mu_{i}\;=T\left(dS^{T}-dS^{M}\right)+\left(S^{T}-S^{M}\right)dT+\sum_{i=1}^{r}\mu_{i}\left(dS_{i}^{C}-dS_{i}^{M}\right)+\sum_{i=1}^{r}\left(S_{i}^{C}-S_{i}^{M}\right)d\mu_{i}\;=TdS^{T}-TdS^{M}+S^{T}dT-S^{M}dT+\sum_{i=1}^{r}\mu_{i}dS_{i}^{C}-\sum_{i=1}^{r}\mu_{i}{dS}_{i}^{M}+\sum_{i=1}^{r}S_{i}^{C}d\mu_{i}-\sum_{i=1}^{r}S_{i}^{M}d\mu_{i}$$

On combining the Gibbs Relation (7) and Euler Relation (9) expressed in differential terms set forth,

$$TdS^{T}-TdS^{M}+\sum_{i=1}^{r}\mu_{i}dS_{i}^{C}-\sum_{i=1}^{r}\mu_{i}dS_{i}^{M}=\;=TdS^{T}-TdS^{M}+S^{T}dT-S^{M}dT+\sum_{i=1}^{r}\mu_{i}dS_{i}^{C}-\sum_{i=1}^{r}\mu_{i}{dS}_{i}^{M}+\sum_{i=1}^{r}S_{i}^{C}d\mu_{i}-\sum_{i=1}^{r}S_{i}^{M}d\mu_{i}$$

hence, the Gibbs–Duhem Relation assumes the following generalized form:

(10) $$S^{T}dT-S^{M}dT+\sum_{i=1}^{r}S_{i}^{C}d\mu_{i}-\sum_{i=1}^{r}S_{i}^{M}d\mu_{i}=0$$

The first member of the above equation can be expressed in terms of (thermal and chemical) generalized entropy property as follows:

(11) $$\left(S^{T}-S^{M}\right)dT+\sum_{i=1}^{r}\left(S_{i}^{C}-S_{i}^{M}\right)d\mu_{i}=0\;\left(S^{G,T}\right)dT+\sum_{i=1}^{r}\left(S_{i}^{G,C}\right)d\mu_{i}=0$$

The superscript “G” stands for “Generalized” and accounts for both thermal and mechanical, or chemical and mechanical, internal energy contributions. Once the total thermal–mechanical contribution is added to the total chemical–mechanical contribution, then the definition of “Generalized” entropy will have been achieved and used in the Generalized Gibbs–Duhem Relation.

In the above Equation (11), the temperature T is the mean value of the inter-particle kinetic energy representing the thermal potential as a component of internal energy and determining the mechanical potential −PV through the frequency of collisions, and transferred to the external system in the form of macroscopic work interaction by means of a weight (or electro-magnetic) process; $\mu_{i}$ constitutes the inter-particle potential energy representing the chemical potential as a component of internal energy resulting in the mechanical potential −PV through the intensity of interactions and transferred in the form of macroscopic work interaction transferred by means of a weight (or electro-magnetic) process. Both thermal potential and chemical potential constitute the two fundamental thermodynamic potentials at microscopic inter-particle level, interacting at macroscopic level, that represent the hierarchical kinematic and geometric configurations of internal energy contributions.

The dualism of thermal potential and chemical potentials constitutes the inherent “emergence” of thermodynamic potentials, even in the special case of an ideal system for which inter-particle potential energy is null. Though, potential energy still exists in the form of repulsive reaction potential energy that is due to kinetic energy transformed at each and every collision, without macroscopic effects on the entire system.

This different form of the Gibbs–Duhem relation resolves the apparent inconsistency in the special case of the isothermal ideal process. In fact, the term $\sum_{i=1}^{r}\left(S_{i}^{C}-S_{i}^{M}\right)d\mu_{i}$ because the system model is ideal and dT=0, remains the only condition to be satisfied since dP no longer appears in the Gibbs–Duhem Relation as expressed in Equation (11). The rationale behind these conclusions can also be found in the dynamics of microscopic particles constituting the macroscopic system. In fact, again considering an isothermal process, the temperature and subsequent intermolecular repulsive interactions at each and every collision are constant, and the differential variations in kinetic potential and chemical potential (due to intermolecular repulsive interactions) are therefore null. In the case of the isobaric process, temperature changes and pressure are constant, hence kinetic potential and chemical potential (due to repulsive interactions at each inter-particle collision) both change along the isobaric process: dT≠0 and consequently $d\mu_{i}\neq 0$. Even in the case of an ideal system, there is a dualism and symmetry of kinetic energy and potential energy among the molecules so that dT>0 and dμ>0 characterizing any state.

Pressure is the mechanical effect of the combined contribution related to thermal interaction and related potential and chemical interaction and related potentials. From this perspective, pressure can be viewed as the outcome of both temperature and chemical potentials of a complex multi-particle system, converted into non-useful and useful work interaction with the external reservoir and with the external weight process, respectively.

Finally, notwithstanding the restrictions assumed for the model adopted, the behavior of the system is coherent with expectations in terms of phenomena and trend of the properties in the general case of real systems and processes where each particle experiences attractive interactions with all others and does not contradict the fundamentals reported in the literature.

The continuous transformation of kinetic energy into potential energy and vice versa occurs at the molecular inter-particle level. Therefore, one form is real, and the other form is apparent, as it derives from the other one, and vice versa.

The configuration of internal energy has a corresponding homology in the different forms of the entropy property, as reflected in the two terms of the second member of the following expressions:

(12) $$S^{T}\left(T,V\right)-S_{0}^{T}=C_{V}\ln\frac{T}{T_{0}}+\bar{R}\ln\frac{V}{V_{0}}$$

(13) $$S^{T}\left(T,P\right)-S_{0}^{T}=C_{P}\ln\frac{T}{T_{0}}-\bar{R}\ln\frac{P}{P_{0}}$$

where heat interaction and work interaction are relating to the two terms of the second member and

(14) $$S^{C}\left(\mu ,V\right)-S_{0}^{C}=C_{n}\ln\frac{\mu }{\mu_{0}}+\bar{R}\ln\frac{V}{V_{0}}$$

(15) $$S^{C}\left(\mu ,P\right)-S_{0}^{C}=C_{P}\ln\frac{\mu }{\mu_{0}}-\bar{R}\ln\frac{P}{P_{0}}$$

where mass interaction and work interaction are relating to the two terms of the second member.

**Figure 1:**
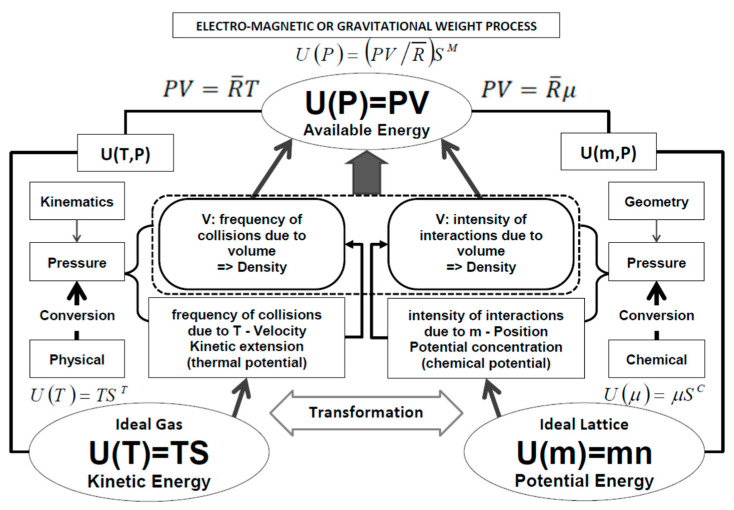
Thermodynamic Potentials.

The configuration of internal energy is determined by thermodynamic potentials (expressed by intensive properties such as temperature, chemical potential, pressure) correlated with each other according to the schema reported in Figure 1—Thermodynamic Potentials.

## 4. Specific Work and Efficiency of Joule and Carnot Cycles

Thermodynamic cycles usually discussed in the literature are those considering temperature as the significant property accounted for to define the cycle efficiency. However, the other intensive property determining the efficiency is the chemical potential as pointed out by Gyftopoulos [17,18]. This correspondence of temperature and chemical potential establishes a symmetry of both Carnot thermal and Carnot chemical ideal cycles which constitute the maximum efficiency cycles between two constant temperatures or chemical potentials imposed by the external systems. As anticipated, the pressure represents an additional thermodynamic potential and, being two opposite process isobaric, then the Joule ideal cycle results as the maximum efficiency cycle between two constant pressures imposed by high pressure and low pressure external systems. The dependence of efficiency on pressure derives from the fact that pressure is a “by-product” of temperature and/or chemical potential being generated by the frequency of collisions and/or intensity of interactions among particles and walls of containers. This dependence is established by thermal and chemical Equation of State and is reflected in the expressions of thermodynamic efficiency and the specific work (representing the net useful work resulting from the balance along a complete closed or open cycle of a given system) [16].

The following sections illustrate the analytical functions of specific work and thermodynamic efficiency depending on temperature, or chemical potential, and pressure for ideal Joule and Carnot direct cycles. The same conclusions remain valid if reference is made to net useful heat representing the specific heat resulting from the inverse cycles by inverting the direction of all the sequential processes that constitute the two conversion cycles.

### 4.1. Joule Cycle: Variational of Specific Work

**Figure 2:**
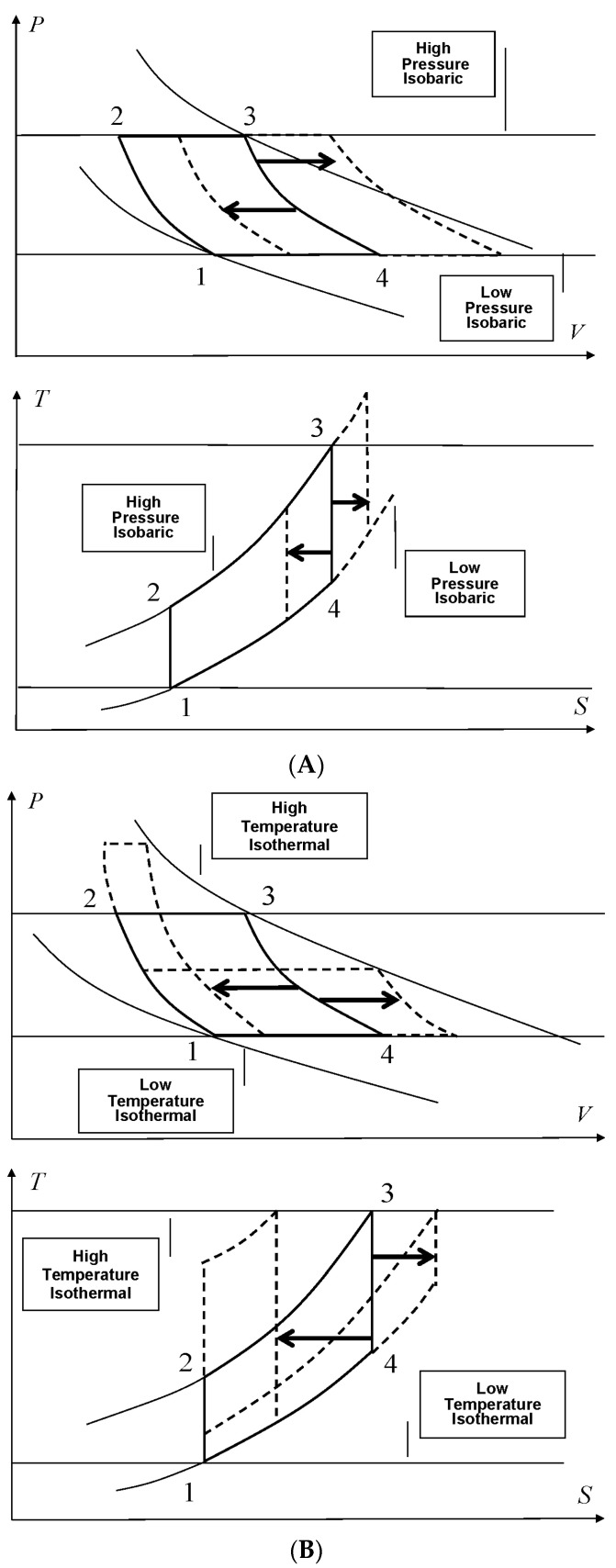

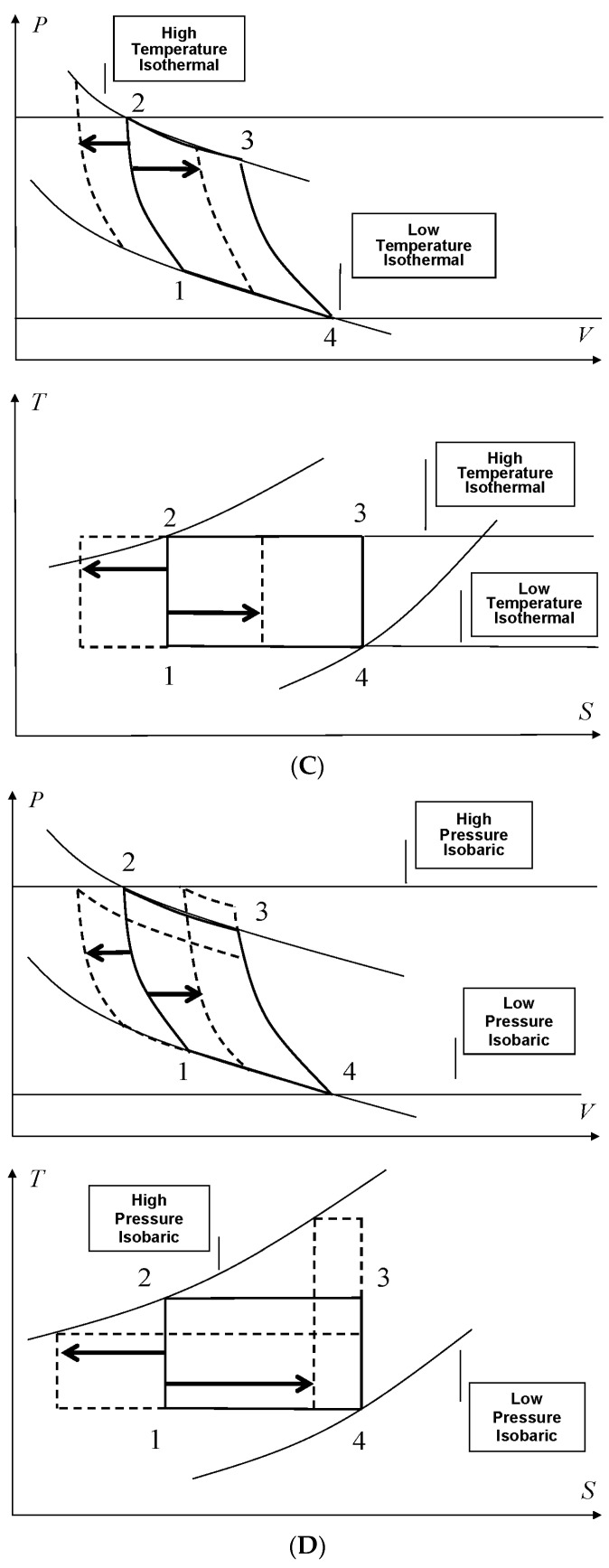
(**A**) Joule cycle: constant extreme pressures and variable higher temperature. (**B**) Joule cycle: constant extreme temperatures and variable higher pressure. (**C**) Carnot cycle: constant extreme temperatures and variable higher pressure. (**D**) Carnot cycle: constant extreme pressures and variable higher temperature.

As far as the Joule ideal cycle is concerned, the specific work over the whole cycle can be calculated by means of the following expression which makes reference to Figure 2A,B; the overall work interaction balance is calculated by the contribution throughout the isobaric processes at higher and lower pressures (the two opposite isodiabatic–adiabatic processes give no contribution to the overall balance):

(16) $$W_{JOULE}^{T}=Q_{ISOBARIC}^{HT}-Q_{ISOBARIC}^{LT}$$

(17) $$W_{JOULE}^{T}=C_{P}\left(T_{3}-T_{2}\right)-C_{P}\left(T_{4}-T_{1}\right)$$

Dividing both members by $C_{P}T_{1}$ the adimensional expressions are obtained:

(18) $$\frac{W_{JOULE}^{T}}{C_{P}T_{1}}=\frac{T_{3}}{T_{1}}-\frac{T_{2}}{T_{1}}-\frac{T_{4}}{T_{1}}+1$$

and considering the relationship temperature-to-pressure along the adiabatic isentropic processes applied to the ratio $T_{3}/T_{2}$ that, by virtue of the property of symmetric cycles, is equal to $T_{4}/T_{1}$:

(19) $$\frac{W_{JOULE}^{T}}{C_{P}T_{1}}=\frac{T_{3}}{T_{1}}-{\left(\frac{P_{2}}{P_{1}}\right)}^{\frac{K-1}{K}}-\frac{\frac{T_{3}}{T_{1}}}{{\left(\frac{P_{2}}{P_{1}}\right)}^{\frac{K-1}{K}}}+1$$

and adopting the symbolic convention: $\tau =\frac{T_{3}}{T_{1}}$, $\pi =\frac{P_{2}}{P_{1}}$, $\lambda =\frac{K-1}{K}$ the above equation assumes the form:

(20) $$\frac{W_{JOULE}^{T}}{C_{P}T_{1}}=1+\tau -\pi^{\lambda }-\frac{\tau }{\pi^{\lambda }}$$

The expression of specific work depends on the extreme temperatures $T_{3}$ and $T_{1}$ and extreme pressures $P_{2}$ and $P_{1}$ of the Joule cycle here analyzed. The specific work resulting from the balance of Joule cycles with the same higher and lower pressures always increases if $T_{3}$ increases, and decreases if $T_{3}$ decreases, while the efficiency remains constant since the ratio of extreme constant pressures remain constant; indeed, the Joule ideal cycle efficiency, depending on pressures only, is expressed as $\eta_{JOULE}^{ideal}=1-1/\pi^{\lambda }$, as shown in Figure 2A.

The consequence of this relationship between specific work and efficiency is the existence of a minimum and a maximum value of the specific work function that can be investigated by means of the derivation of the analytical non-dimensional specific work function with respect to τ:

(21) $$\frac{d}{d\tau }\left(\frac{W_{JOULE}^{T}}{C_{P}T_{1}}\right)=\frac{d}{d\tau }\left(1+\tau -\pi^{\lambda }-\frac{\tau }{\pi^{\lambda }}\right)=1-\frac{1}{\pi^{\lambda }}=\frac{\pi^{\lambda }-1}{\pi^{\lambda }}$$

and, equalizing to zero:

(22) $$\frac{d}{d\tau }\left(\frac{W_{JOULE}^{T}}{C_{P}T_{1}}\right)=0⟹\frac{\pi^{\lambda }-1}{\pi^{\lambda }}=0⟹\pi^{\lambda }=1⟹\pi =1$$

hence, the specific work is maximum between the extreme higher and lower temperature variations if, and only if, the cycle’s extreme higher and lower pressures are equal. Thus, the equality of extreme pressures determines the degeneration of the cycle into an isobaric process occurring in two opposite directions and, subsequently, no maximum or minimum values exist for the derivative of the specific work in the monotone non-dimensional expression.

Vice versa, if $T_{1}$ and $T_{3}$ (or τ) are fixed and $P_{1}$ and/or $P_{2}$ (or π) change in the direction of increasing or decreasing volume, as described in Figure 2B, then the specific work function in Equation (20) is no longer monotone. Indeed, if $P_{1}=P_{2}$ then π=1 and the specific work is null.

At the same time, if $\frac{T_{3}}{T_{1}}={\left(\frac{P_{3}}{P_{1}}\right)}^{\frac{K-1}{K}}={\left(\frac{P_{2}}{P_{1}}\right)}^{\frac{K-1}{K}}$ or if $\pi^{\lambda }=\tau$ which can be written as $\pi =\tau^{\frac{1}{\lambda }}$, then the specific work is once again null. According to Rolle’s theorem, valid for a continuous and derivable function, a relative maximum or minimum value is positioned in between the two pressure extreme values where the function itself is null; this value satisfies the following variational condition:

(23) $$\begin{array}{l} \frac{d}{d\pi }\left(\frac{W_{JOULE}^{T}}{C_{P}T_{1}}\right)=\frac{d}{d\pi }\left(1+\tau -\pi^{\lambda }-\frac{\tau }{\pi^{\lambda }}\right)\\ =\lambda \pi^{\lambda -1}+\lambda \tau \pi^{-\lambda -1}=0 \end{array}$$

from which, explicating the pressures ratio:

(24) $$\pi =\tau^{\frac{1}{2\lambda }}$$

It is noteworthy that if τ=1 then π=1 necessarily and this implies that the cycle degenerates into one single point.

Figure 2B shows how the geometry of the Joule cycle changes with a variation in extreme higher pressure while extreme temperatures of the cycle itself remain constant.

To conclude this analysis, the maximum specific work of a Joule ideal cycle, depending on the variation in extreme pressures ratio, is positioned in correspondence to the mean value of pressures ratio 1/2λ in between and at the same distance from extreme pressures with null specific work and temperatures kept constant at the pressure’s variation.

### 4.2. Carnot Cycle: Variational of Specific Work

Focusing on the Carnot ideal cycle, with a similar rationale already adopted for the Joule cycle, the specific work variation is now analyzed. The overall work balance is calculated considering the contribution throughout the isothermal processes at higher and lower temperatures where heat and work interactions occur. The two adiabatic processes make available no contribution as the cycle is symmetric, and the pressures ratio is the same, thus determining an equal and opposite contribution to overall work interaction balance. Figure 2C,D represent similar variations as in the case of Joule cycle:

(25) $$W_{CARNOT}^{T}=Q_{ISOTHERMAL}^{HT}-Q_{ISOTHERMAL}^{LT}=-\left(P_{3}V_{3}\ln\frac{P_{2}}{P_{3}}-P_{4}V_{4}\ln\frac{P_{1}}{P_{4}}\right)$$

The validity of thermal state equation $PV=n\bar{R}T$ is inherent in the definition of ideal gas isothermal reversible processes, and therefore it can be used to obtain the following:

(26) $$W_{CARNOT}^{T}=-\left(n\bar{R}T_{3}\ln\frac{P_{2}}{P_{3}}-n\bar{R}T_{4}\ln\frac{P_{1}}{P_{4}}\right)$$

to treat the above function in non-dimensional terms, the expression of $W_{CARNOT}^{T}$ is entirely divided by the term $n\bar{R}T_{1}=n\bar{R}T_{4}$ and, since $T_{1}=T_{4}$ being located on the same isothermal process, the above equation becomes:

(27) $$\frac{W_{CARNOT}^{T}}{n\bar{R}T_{1}}=-\frac{T_{3}}{T_{1}}\ln\frac{P_{2}}{P_{3}}+\ln\frac{P_{1}}{P_{4}}$$

and multiplying the two arguments of both logarithm terms by $\frac{P_{4}}{P_{4}}$ and $\frac{P_{2}}{P_{2}}$, respectively, the following is obtained:

(28) $$\frac{W_{CARNOT}^{T}}{n\bar{R}T_{1}}=-\frac{T_{3}}{T_{1}}\ln\frac{P_{2}}{P_{3}}\frac{P_{4}}{P_{4}}+\ln\frac{P_{1}}{P_{4}}\frac{P_{2}}{P_{2}}$$

considering that, for the reversible adiabatic processes (opposite isodiabatics) connecting the isothermal ones of the cycle, the following relationships are applicable: $\frac{T_{3}}{T_{4}}={\left(\frac{P_{3}}{P_{4}}\right)}^{\frac{K-1}{K}}\Rightarrow \frac{P_{3}}{P_{4}}={\left(\frac{T_{3}}{T_{4}}\right)}^{\frac{K}{K-1}}$ and $\frac{T_{2}}{T_{1}}={\left(\frac{P_{2}}{P_{1}}\right)}^{\frac{K-1}{K}}\Rightarrow \frac{P_{2}}{P_{1}}={\left(\frac{T_{2}}{T_{1}}\right)}^{\frac{K}{K-1}}$ and substituting in the above equation:

(29) $$\frac{W_{CARNOT}^{T}}{n\bar{R}T_{1}}=-\frac{T_{3}}{T_{1}}\ln\left[\frac{P_{2}}{P_{4}}{\left(\frac{T_{3}}{T_{4}}\right)}^{-\frac{K}{K-1}}\right]+ln\left[\frac{P_{2}}{P_{4}}{\left(\frac{T_{2}}{T_{1}}\right)}^{-\frac{K}{K-1}}\right]$$

considering the thermodynamic diagrams and the defined states $T_{1}=T_{4}$ and $T_{2}=T_{3}$, respectively, then:

(30) $$\frac{W_{CARNOT}^{T}}{n\bar{R}T_{1}}=-\frac{T_{2}}{T_{4}}\ln\left[\frac{P_{2}}{P_{4}}{\left(\frac{T_{2}}{T_{4}}\right)}^{-\frac{K}{K-1}}\right]+ln\left[\frac{P_{2}}{P_{4}}{\left(\frac{T_{2}}{T_{4}}\right)}^{-\frac{K}{K-1}}\right]$$

making reference to above equalities of temperatures and pressures and the already stated symbolic convention of the properties of symmetric cycles: $\tau =\frac{T_{2}}{T_{4}}$, $\pi =\frac{P_{2}}{P_{4}}$, $\lambda =\frac{K-1}{K}$ it follows that the previous equation can be simplified in the form:

(31) $$\frac{()W_{CARNOT}^{T}}{n\bar{R}T_{1}}=-\tau \ln\left[\pi {\left(\tau \right)}^{-\frac{1}{\lambda }}\right]+ln\left[\pi {\left(\tau \right)}^{-\frac{1}{\lambda }}\right]$$

This expression of the specific work is derived for the Carnot cycle properties and is formally different with respect to the expression with the same meaning calculated for the Joule cycle; for both expressions, extreme temperatures (or extreme chemical potentials) and pressures are intensive properties governing processes and variations. Hence, if the extreme cycle temperatures are equal, the specific work always increases if $P_{3}$ increases and/or $P_{1}$ decreases as reported in Figure 2C.

The dependance of specific work of the Carnot cycle is demonstrated by deriving the value of the cycle’s extreme pressures determining the maximum value of the specific work for the Carnot cycle with a similar procedure already adopted of the Joule cycle, thus resulting that:

(32) $$\frac{d}{d\pi }\left(\frac{W_{CARNOT}^{T}}{n\bar{R}T_{1}}\right)=\frac{d}{d\pi }\left(-\tau \ln\left[\pi {\left(\tau \right)}^{-\frac{1}{\lambda }}\right]+ln\left[\pi {\left(\tau \right)}^{-\frac{1}{\lambda }}\right]\right)\;=-\tau \cdot {\left(\tau \right)}^{-\frac{1}{\lambda }}\cdot \frac{1}{\pi {\left(\tau \right)}^{-\frac{1}{\lambda }}}+{\left(\tau \right)}^{-\frac{1}{\lambda }}\cdot \frac{1}{\pi {\left(\tau \right)}^{-\frac{1}{\lambda }}}=\left(1-\tau \right)\cdot \left(-\frac{\tau^{-\frac{1}{\lambda }}}{{\pi \tau }^{-\frac{1}{\lambda }}}\right)=\frac{1-\tau }{\pi }$$

Equalizing to zero the following result is obtained:

(33) $$\frac{d}{d\pi }\left(\frac{W_{CARNOT}^{T}}{n\bar{R}T_{1}}\right)=\frac{1-\tau }{\pi }=0\;\Rightarrow \tau =1$$

It can be deduced that, with respect to the Joule cycle, swapping temperature with pressure implies that the Carnot cycle is characterized by a maximum value of the specific work if and only if the cycle’s extreme temperatures are equal and the cycle itself degenerates into an isothermal process executed in the two directions.

Vice versa, if the Carnot cycle’s extreme pressures are fixed so that π is constant and the cycle’s extreme temperatures change by means of ratio τ as shown in Figure 2D, it is possible to search for a value of τ maximizing the specific work balance.

Assuming that $\frac{W_{CARNOT}^{T}}{n\bar{R}T_{1}}=0$ Equation (31) becomes:

(34) $$\tau \ln\left[\pi {\left(\tau \right)}^{-\frac{1}{\lambda }}\right]=ln\left[\pi {\left(\tau \right)}^{-\frac{1}{\lambda }}\right]$$

which is verified if τ=1 or if $\pi {\left(\tau \right)}^{-\frac{1}{\lambda }}=1$ or if $\tau =\pi^{\lambda }$ or if $\pi =\tau^{\frac{1}{\lambda }}$ and, as will be noted, this last expression equals the corresponding one derived from the Joule cycle.

The equality of the two expressions proves the symmetry of the effect due to temperature ratios and pressure ratios that render null the specific work of the Carnot cycle and the Joule cycle, respectively, and hence the role of temperature and pressure in the two cycles. This result also demonstrates the symmetry of variational for specific work calculated over the whole cyclic process based on the symmetry of both cycles compared through intensive properties.

Now that, also in the Carnot cycle, the specific work is null for two different values of property τ then, by virtue of Rolle’s theorem, there must be at least one value of τ where the specific work function reaches a relative extremum. This value can be calculated by deriving the non-dimensional expression of the specific work, reported in Equation (31), with respect to τ:

(35) $$\frac{d}{d\tau }\left(\frac{W_{CARNOT}^{T}}{n\bar{R}T_{1}}\right)=\frac{d}{d\tau }\left\{-\tau \ln\left[\pi {\left(\tau \right)}^{-\frac{1}{\lambda }}\right]+ln\left[\pi {\left(\tau \right)}^{-\frac{1}{\lambda }}\right]\right\}\;=\frac{d}{d\tau }\left\{\left(1-\tau \right)\cdot ln\left[\pi {\left(\tau \right)}^{-\frac{1}{\lambda }}\right]\right\}=0$$

which becomes, after calculating the derivative:

(36) $$\frac{\tau -1}{\tau \lambda }-\ln\pi \tau^{\frac{1}{\lambda }}=0=>\;\frac{\tau -1}{\tau }=\ln\frac{\pi }{\tau }=>\;\frac{\tau -1}{\tau }+\ln\tau =\ln\pi$$

The last expression provides, for any fixed value of π, the corresponding value of τ to determine the specific work of the Carnot cycle determining the extreme value of its function. If π=1 in the previous equation, then τ=1; this result implies, in analogy with the result obtained with a similar procedure for the Joule cycle, that the Carnot cycle degenerates into one single point.

Figure 2D shows the geometric variation in the Carnot ideal cycle for the variation in extreme temperatures while extreme pressures remain constant.

In particular, whatever the Carnot cycle between two constant extreme pressures, the work interaction $dW_{ISOTHERMAL}^{HT}$ for an infinitesimal cycle, is characterized by a maximum value of the specific work that tends to decrease if the adiabatic process of this infinitesimal cycle is adjusted in the direction of the highest or lowest volume range and consequently the specific work decreases in both cases on the basis of the conclusions reached in the previous sections. Thus, a Joule cycle can be accomplished through an infinite series of infinitesimal Carnot cycles with the result that Joule cycle maximum specific work remains constant if isobaric processes are finite. Vice versa, whatever the Joule cycle between two constant extreme temperatures, the same conclusion is validated for the Carnot cycle.

The rationale and the thesis achieved so far remain valid and applicable to Carnot cycles determined by extreme chemical potentials in lieu of temperatures also considering that pressure is the result of temperature and/or chemical potentials characterizing any system in any state.

## 5. Dualisms and Symmetries in Generalized Exergy and Entropy Properties

The variational approach, adopted to search for the maximum performance in terms of specific work interaction of Carnot and Joule cycles, has resulted in a symmetric geometry of the two cycles with respect to temperatures, or chemical potentials, compared to the pressures as thermodynamic potentials representing the reference entities of symmetry. The variation in extreme pressure differences determines the variation in the specific work that in effect represents an indirect consequence of the mechanical thermodynamic efficiency; hence, for thermal–mechanical or chemical–mechanical cycles, the specific work is associated with the thermodynamic efficiency of the mechanical aspect only. Considering that Carnot and Joule cycles constitute the rationale for expressing thermal, chemical, and mechanical exergy definitions, the optimization of efficiency, jointly with the specific work and specific heat, or specific work and specific mass (termed as specific interactions) can be retrieved in the definition and physical meaning of the exergy property. In fact, even in the special case of ideal systems and reversible processes, efficiency and specific interactions depend on, and are affected by, the system’s high and low temperatures ratio (or high and low chemical potentials ratio) and by high and low pressures ratio, with inverted effects in the Carnot cycle with respect to the Joule cycle. In a different perspective, the dualism of temperature and pressure, or chemical potential and pressure, has a symmetric effect considering the behavior of Carnot and Joule cycles in the sense that pressure is the dual thermodynamic potential with respect to temperature or chemical potential, while the pressure determines a symmetric geometric configuration of Carnot and Joule cycles characterizing the formulation of exergy property. Therefore, as the pressure constitutes a thermodynamic potential determining the mechanical exergy, hence the mechanical entropy associated with specific volume (or density) can be derived as a property of the generalized exergy including the mechanical exergy component [17,18].

The definition of generalized exergy implies corollaries extended to thermal and chemical thermodynamic potentials. Moreover, the mechanical thermodynamic potential, depending on internal pressure or density, constitutes an additional component with respect to temperature and chemical potentials. Nevertheless, a caveat needs to be clarified in this regard since pressure is a derived property because it is the consequence of temperature, or chemical potentials, or the combination of both, through the specific volume corresponding to the density determining the frequency and intensity of interactions among particles and between particles and containing walls. In terms of formal expressions, the thermal internal energy $TS^{T}$, and/or the chemical internal energy $\sum_{i=1}^{r}\mu_{i}S_{i}^{C}$, corresponds to the mechanical internal energy PV, even though an isothermal process and/or an isopotential process has different meaning with respect to an isobaric process. An isothermal process at constant temperature, and/or an isopotential process at constant chemical potential, occurs at a constant internal energy resulting from the contribution of energy associated with each and every particle. While an isobaric process, at constant pressure, does not correspond to a process at constant internal energy as the pressure is not associated with particles since resulting from the composition of multiple actions exerted through the volume as extensive property. Hence, the internal energy changes, and for example, it increases because an increase in temperature, and/or chemical potentials, is needed to keep the pressure constant while the volume increases, and the opposite for decrease. In different terms, in case of volume increase, the constant pressure requires an increase in temperature, and/or chemical potentials, implying an increase in thermal entropy, and/or chemical entropy (due to temperature, and/or chemical potentials, and volume increase) and a subsequent increase in thermal internal energy $U^{T}=TS^{T}$, and/or chemical internal energy $\sum_{i=1}^{r}\mu_{i}S_{i}^{C}$ that in turn determines an increase in mechanical internal energy $U^{M}=PV$ as a contribution under an additional, and different, physical form. Indeed, the total amount of heat interaction input, and/or mass interaction input, is subdivided into thermal and mechanical forms, and/or chemical and mechanical forms, of internal energy because a portion of the thermal internal energy, and/or chemical internal energy, is transformed into mechanical internal energy at constant pressure, and/or constant chemical potentials, and increasing volume, and vice versa for decreasing interactions and properties.

In consideration of the role assumed by pressure and mechanical internal energy, it may be argued that the definition of exergy property should be affected by, and founded on, the two concepts of dualism and symmetry, characterizing Carnot and Joule cycles. Indeed, dualisms are demonstrated through the correlation of Carnot and Joule cycles with temperature, or chemical potentials, and pressure, while the symmetries are based on the relationship of thermal, chemical and mechanical thermodynamic potentials with thermodynamic efficiency and specific work constituting the fundamental characteristics of any cyclic process of equilibrium and non-equilibrium states.

The generalized formulation of the exergy property accounts for the two component processes, isoentropic and isovolumic, that, on a bi-dimensional thermodynamic plan, such as pressure–volume or temperature–thermal entropy (or chemical potential–chemical entropy), can connect any pair of points associated with thermodynamic states of any system. These processes are characterized by only one type of interaction, that is, work interaction for isoentropic (adiabatic and non-permeable and reversible) and heat interaction, or mass interaction, for isovolumic. Both interactions can be measured in terms of exergy represented by the amount of work interaction withdrawn by means of heat-to-work and/or mass-to-work (direct) conversion process and by the amount of heat interaction and/or mass interaction withdrawn by means of work-to-heat and/or work-to-mass (indirect) conversion process. Considering that exergy property depends on initial and final states only by definition, hence it can be calculated through any couple of thermodynamic elaborations, such as the very isoentropc–isovolumic, among whatever other couple of processes. Using the isoentropic implies that work interaction must be evaluated in terms of energy conversion, or exergy input or output, exchanged with the external system. It is evident that the work interaction along an isoentropic (adiabatic) process becomes susceptible to conversion efficiency variation depending on the pressure and the mechanical entropy correlated to volume or density. This approach leads to generalizing the expression of exergy property thus resulting from the contribution of interactions with the external useful system and non-useful reservoir:

(37) $${EX}^{G}={EX}_{REV}^{T}+{EX}_{REV}^{C}+{EX}_{REV}^{M}=W_{REV}^{T}+W_{REV}^{C}+Q_{REV}+M_{REV}\;=W_{REV}^{T-CONVER}+W_{REV}^{T-TRANSF}+W_{REV}^{C-CONVER}+W_{REV}^{C-TRANSF}\;+Q_{REV}^{CONVER}+Q_{REV}^{TRANSF}+M_{REV}^{CONVER}+M_{REV}^{TRANSF}$$

where (i) the superscript “CONVER” is related to input interaction converted into output useful interaction and depends on reversible or irreversible energy conversion; and (ii) “TRANSF” is the non-useful output interaction, transformed from the non-converted portion of available energy, constituting the energy loss transferred to the external non-useful reservoir.

The above formulation of generalized exergy embeds the concept of “exergy of exergy” [17,18] proposed to define the “counter-evaluation” of work interaction to evaluate the mechanical exergy by means of heat and mass interactions, with the opposite approach with respect to thermal and chemical exergies. Considering the entropy–exergy relationship underpinning the definition of entropy property components, the concept of “entropy of entropy” is derived. It is noteworthy that the exergy property takes into account the internal energy or enthalpy in the sense that exergy loss constitutes the amount of internal energy reversibly released to the external system, or a portion of internal system behaving as a reservoir, hence non-useful; this loss is moved by thermodynamic potentials levels of the internal system higher than the reservoir; the exergy loss is an additional contribution other than the exergy destruction constituting the amount of internal energy irreversibly dissipated among internal portions of the system, or among portions of internal and external systems, with a subsequent depletion of the generalized and non-generalized adiabatic availability or available energy. Consequently, the overall generalized efficiency is expressed as the product of energy efficiency and exergy efficiency: $\eta^{G}=\eta^{EN}\cdot \eta^{EX}$.

## 6. Necessity and Sufficiency of Stable Equilibrium and Equality of Potentials

**Figure 3:**
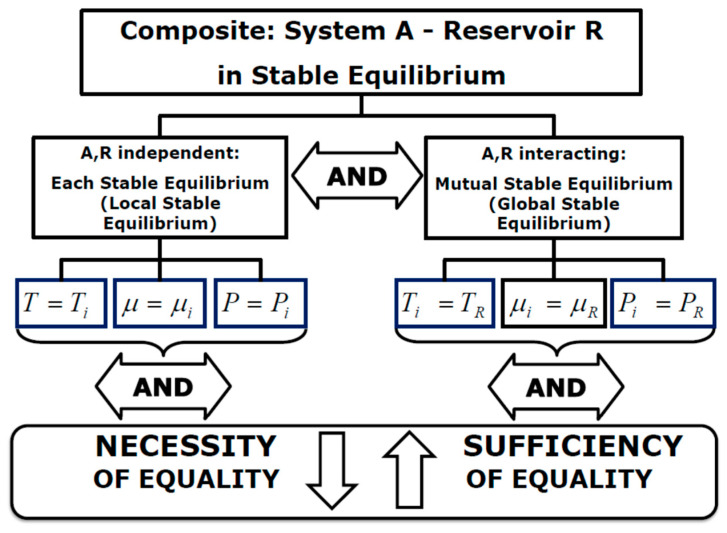
Implication of necessity and sufficiency of stable equilibrium.

The central concept of the definition of the Second Law, developed according to MIT line of thought, is stated in terms of existence and uniqueness of stable equilibrium [5]. Founded on this statement, the definition of the entropy property is inferred from the concepts of energy and available energy (derived from the adiabatic availability or non-interaction availability) and represents a corollary of the Second Law. On this basis, the entropy property is an intermediate step to demonstrate that temperature, chemical potentials, and pressure behave as thermodynamic potentials [5]. The proofs are elaborated from the concept of the impossibility of a Perpetual Motion Machine of the Second Kind (PMM2) but arguing the validity of stable equilibrium for equality of thermodynamic potentials in logical terms of sufficiency only, thus disregarding the necessity. Though, pressure represents an additional thermodynamic potential that should be accounted for in stable equilibrium. Notwithstanding pressure and volume properties are constrained to the temperature, or to the chemical potentials, by means of Thermal Equation of State, or Chemical Equation of State [16], the product PV is independently determined by variations in pressure and volumes while complying with the overall equality of thermodynamic potentials to ensure the stable equilibrium. The opposite logical relationship, from equality of potentials to stable equilibrium, consists of the very necessity of the stable equilibrium. Hence, combining the two logical relationships, equality of thermodynamic potentials ends up being a necessary and sufficient condition for stable equilibrium, and vice versa, the stable equilibrium must be a necessary and sufficient condition for the equality of potentials [19,20]. The schema of the biunivocal logical implication of stable equilibrium and equality of potentials is represented in the Figure 3. This logical relationship has to be valid to confirm the generality of the Second Law expressed in terms of “existence and uniqueness of stable equilibrium” as stated by Gyftopoulos and Beretta [5] and corroborates the need of the mechanical entropy, as an additional component of the generalized entropy, to achieve the full validity of the Second Law statement in terms of necessity and sufficiency of stable equilibrium or necessity and sufficiency of equality of potentials. This biunivocal logical implication, and the subsequent logical paradigm represented in the following Figure 3, is founded on the coexistence of local and global equality of thermodynamic potentials correlated to the local and global stable equilibrium needed for the bi-directional necessity and sufficiency logical relationships [19,20].

## 7. Thermodynamic and Informational Aspect of Extrema Principles

Among outstanding achievements, and subsequent corollaries, of the Unified Quantum Theory of Mechanics and Thermodynamics, the inherent physical ultimate essence of irreversibility of real phenomena lies in the intrinsic quantum characteristics of any physical entity [21,22]. All principles can be retrieved and proved at a microscopic or infinitesimal scale and non-equilibrium phenomena are the fundamental basis of irreversible real processes [23,24]. Mathematical variational methods are used in physics to seek maximum or minimum extrema of functions translating phenomena. Non-equilibrium processes are characterized by maximum or minimum generation or production of characteristic properties, such as entropy and exergy, that provide a rational description of the root causes of degradation and creation phenomena within systems constraints by external conditions. As far as entropy property extrema principles are concerned, the maximum entropy production at a microscopic level is described by the Steepest-Entropy-Ascent approach [25,26]. A rigorous review of extrema principles, and reciprocal relationships, is reported in the literature [27,28].

Although information is not implied in the fundamental nature of physical entities and related phenomena, a relation among physical and informational aspects of systems at equilibrium and non-equilibrium states has been observed and demonstrated [29]. This perspective is valid considering the physical aspect; nevertheless, the content of information and the degree of self-organization is inherently associated with thermodynamic states and non-equilibrium processes. In this regard, the Landauer Principle assumes that physical and informational aspects are correlated; if not, they represent two different expressions of the same physical–informational ultimate essence of any entities [30,31]. Nevertheless, recent research argues that physical processes of information transduction can be thought of as idealized until a reduction in the energetic degradation caused by irreversible phenomena of physical devices used to store and transmit information. The underpinning fundamental relationship between physical and informational character is established by the Landauer theorem correlating energy and information properties and their degradation processes.

In this regard, it seems that the “physical nature of information” characterizes the thermodynamic–informational dualism in analogy with all general laws governing any system in the universe [32,33].

An interesting evolution is represented by the set of “extrema principles” describing the dynamics of non-equilibrium by means of entropy and exergy properties variations, though the Information Theory includes extrema principles relating to informational properties hence representing a set of concepts complementary to those principles stated and valid for strictly physical systems. There is a difference between entropy extrema principles and exergy extrema principles due to the very meaning of the two properties. The more extended meaning of exergy is that this property accounts for both energy reversible, and non-dissipative, losses and energy irreversible dissipative degradation, while entropy focuses on irreversible dissipation only. This difference suggests establishing a paradigm, extended to both thermodynamic and informational aspects, to attempt a unification of all extrema principles in the direction of a more general perspective that can be unified in terms of a dynamical relationship between physical and informational essential nature of ultimate causes of change in any system in any state. This unification should rely on the generalization of thermodynamic properties and the associated Highest-Generalized-Entropy Principle being a consequence of the theorem of necessity and sufficiency above argued [20].

The extrema principles provide a rationale to assess the direction of phenomena with respect to the stable equilibrium along far-from-equilibrium processes. In particular, exergy is not used to improve processes rather than to orient evolutions of individuals along metamorphosis or species along evolution.

### 7.1. Complexity and Self-Organization

The concept of complexity, developed through the method of complexity and complex thought, is the masterpiece of a French philosopher and epistemologist, namely Edgar Morin, who has conceived and developed the theory of complexity described in several textbooks constituting “La Methode” (“The Method”), among which the following ones overarch a perspective of science epistemology: “The Nature of Nature”, “The Life of Life”, “The Knowledge of Knowledge”.

The complexity of thermodynamic and informational processes can be retrieved in living organisms, in the number of metabolic biochemical reactions within a cell rise to about 4000. This is a measure of the complexity of processes ensuring all functions at molecular, supramolecular and cellular level.

The concept of complexity is founded on the existence of different macroscopic, mesoscopic and microscopic hierarchical levels constituting all systems in their most general disposition [20]. Those hierarchical levels are related to each other so that any type of relationship governs emergence of systems behavior. The complexity, through hierarchical levels from microscopic to macroscopic, overarches both physical and informational aspects of all entities and properties. Self-organization is originated by coexistence and the correlation among hierarchical levels and inherent aspects constituting the ultimate elementary and minimal essence of systems and their constituting particles. Hence, self-organization is a characteristic property of complexity and complex systems and their emerging phenomena. In this regard, it can be assumed that the correspondence and the complementarity between thermodynamics and information remains linear until complexity emerges, and self-organization occurs, hence implying the transition from linearity to non-linearity of ontogenetic and phylogenetic processes of individuals and living species.

Self-organization is a consequence of thermodynamic and information extrema principles emerging from non-equilibrium processes compatible with constraints imposed by system constituents and parameters [34].

Complex systems exhibit the capability of self-organizing along non-equilibrium of far-from-equilibrium processes in the frame of principles that can be summarized as follows according to the literature [35,36,37]: (i) the system undergoes metamorphic changes from less to more inter-correlated relationships among constituting elements in the direction of a final configuration achieved by means of available degrees of freedom; (ii) only internal and inter-particle interactions determine dynamical phenomena and the guidance along microscopic processes within the set of constraints imposed by the external system; (iii) global or holistic dynamics governs processes prevailing on local and reductionistic paradigms extending the set on internal restrictions; (iv) statistical phenomena affect a large number of elements requested to interact and contribute to changes, hence amplifying the effects on the overall system. This frame of axiomatic theoretical and experimental facts represents the context where the characters of living systems in the conception of Jacques Monod [38] can be retrieved and corroborated. Monod outlined general processes that living systems only are capable to accommodate: (i) teleonomy: each and every individual organism embeds its own design plan codified under informational code in the nucleic acids of any cell; (ii) invariant reproduction: within any living species or population, individuals reproduce themselves into other individuals with same genotypic and phenotypic characteristics of the species or population their parents belong to; (iii) internal driving forces or thermodynamic potentials and internal interactions among constituting particles are those responsible for embryonal and individual development or metamorphosis, while external interactions with environment do not contribute.

### 7.2. Constructal Theory and Autopoiesis of Living Organisms

Self-organization can be envisaged as an extension of the concept of design, and the opposite hetero-organization is the result of the combination of external actions on non-organized constituting elements. The design and the realization of any non-living system is founded on a sequence of assembling rules, translating scientific laws, leading to building up an artifact with predefined characteristics. The design represents the “external informational exergy” receiving a flow of informational energy input and subsequently producing an organized system characterized by the associated informational energy output and consisting of the constrained degrees of freedom pertaining to each and every component part.

In the frame of extrema principles, and specifically on the basis of the minimum entropy generation principle at macroscopic level, the Constructal Theory has been devised and developed by Bejan [39,40,41,42,43] to provide an overarching perspective and a rationale of complex morphologies development and self-organizing processes. This approach assumes the minimization of entropy generation of dissipation phenomena, hence increasing the access to currents of mass flow from a single-point source to a multi-point sink in mass transport systems configurations and morphologies, though this approach has been extended to overcome limitations by the maximum entropy generation approach. To demonstrate the validity of this extension, Grazzini and Lucia have developed a theoretical and experimental approach to complex phenomena of systems characterized by self-organizing capabilities [44,45].

This extended principle, in its generality, provides a perspective on any kind of system, including living organisms.

In the framework of the Constructal Theory, and differently from hetero-organizing systems, the self-organization of a non-living system derives from an “a priori” design based on a logical sequence of rules governed by scientific laws; this set of rules constitutes the “internal informational exergy” of an open (or closed) system. The informational energy input is elaborated within the set of thermodynamic and informational phenomena, and the inherent informational exergy with the result of a hetero-organization and a constructal set of processes achieving a specific and optimized design of the system. Instead, the teleonomy of living systems is mediated through nucleic acids and, in particular, the Desossirybo-Nucleic Acid, or DNA, constituting the internal design or the “internal informational exergy” of an open (or closed) system, such as any living organism; the informational energy input, elaborated through the informational exergy, generates the self-organization, or autopoiesis, at any hierarchical level of the morphologic and functional configuration.

Living organisms undergo a phylogenetic evolution led by stochastic probabilistic mutations in nucleotide sequences of the genome and by the symmetry breaking in evolutive options selections and exclusions. This is the basic mechanism of species formation processes (such as allopatric, peripatric, parapatric and sympatric), conveying morphologies and phenotypic characters of living organisms. Most successful options are those underpinned by more complex organization being the organization as the entity making “the whole system as more than the sum of its constituting elementary parts”, as enunciated by Aristotle. Autopoiesis represents a self-organizing and self-regenerating set of chemical and informational phenomena and can be considered an evolutionary or metamorphic paradigm of living organisms governed by their own internal complexity along non-equilibrium processes. The capability of self-organization, emerging from systems complexity, should be considered as rooted in the twofold nature of any entity pertaining to the “physis”, that is, the thermodynamic and informational aspects governing any system in any state. From this perspective, autopoiesis would become an emergence and a consequence of complex systems dynamics undergoing coexisting degradation and reconstruction, or regeneration, in far-non-equilibrium homeostatic or transitory conditions. This analogy between phylogenetic evolution and ontogenetic metamorphosis of living organisms establishes a set of governing laws, the validity of which has its primary prerequisite in the complexity inherent to systems and the subsequent self-organizing emerging capabilities.

## 8. Conclusions

A unitary perspective on thermodynamic and informational aspects of physical entities and related phenomena has led us to discuss foundations and envisage future developments of the theoretical method beyond applications. From a different point of view, Carnot and Joule cycles can be derived as corollaries of the Second Law and provide dual and symmetric properties. This paradigm has been extended to thermodynamic potentials moving any process and characterized by the logical symmetry of necessity and the sufficiency of potentials equality for stable equilibrium, and vice versa. Moreover, thermal, chemical and mechanical potentials are related to the generalized entropy and exergy properties, both determined by the extrema principles governing non-equilibrium processes. In the overarching perspective of thermodynamic and informational aspects of multi-layer complex systems with self-organizing capabilities, special attention is deserved by the ever-growing field of biotechnology [27,44,45]. Metabolic pathways, through anabolic or catabolic processes, can be studied with a paradigm based on the twofold thermodynamic and informational standpoint. In this regard, the application of the exergy method provides, and at the same time needs, a rigorous and rational schema to analyze living organisms and all metabolic processes, bioenergetic transductions and the complex set of pathways involving somatic and enzymatic proteins synthesis. Indeed, a focus on the central dogma of biology provides the main direction of any process; that is, the chemical and informational logical sequence from nucleic acids and DNA, through transcription into all types of RNA constituting the transcriptome, up to the translation into all somatic and enzymatic proteins constituting the proteome. Any physical operation and chemical reaction are governed by the concurrence of the First and Second Laws and for this very reason exergy, and its components, is the most suitable property to allow a deep insight into chemical–physical mechanisms underpinning all processes affecting living organisms.

Considering the extended environment composed of lithosphere, hydrosphere, atmosphere and biosphere, the analysis of circularity and energy optimization of any set of processes can be evaluated by means of mass–energy–exergy–information lifecycle assessment approaches accounting for any contribution and interaction with the environment. Nowadays, this approach is demanded in industry businesses to foster technology transition in compliance with environmental protection.

## Data Availability

No data are generated or elaborated in this research, therefore data sharing is not applicable.
